# Microbial communities in placentas from term normal pregnancy exhibit spatially variable profiles

**DOI:** 10.1038/s41598-017-11514-4

**Published:** 2017-09-11

**Authors:** Lindsay A. Parnell, Catherine M. Briggs, Bin Cao, Omar Delannoy-Bruno, Andrew E. Schrieffer, Indira U. Mysorekar

**Affiliations:** 1 Department of Obstetrics and Gynecology, Washington University School of Medicine, 660 South Euclid Avenue, St. Louis, MO 63110 USA; 2 Department of Genetics, Washington University School of Medicine, 660 South Euclid Avenue, St. Louis, MO 63110 USA; 3 Department of Pathology and Immunology, Washington University School of Medicine, 660 South Euclid Avenue, St. Louis, MO 63110 USA

## Abstract

The placenta is the principal organ nurturing the fetus during pregnancy and was traditionally considered to be sterile. Recent work has suggested that the placenta harbours microbial communities, however the location and possible function of these microbes remain to be confirmed and elucidated. Here, we employed genomic DNA sequencing of multiple variable (V) regions of the bacterial 16S ribosomal gene, to interrogate microbial profiles in term pregnancies, from the basal plate, which is in direct contact with maternal uterine, endothelial, and immune cells; placental villi, which are bathed in maternal blood, and fetal membranes, which encapsulate the amniotic cavity. QIIME, R package “Phyloseq” analysis was used to assess alpha and beta diversity and absolute abundance of the 16S rRNA gene per location. We demonstrate that (1) microbiota exhibit spatially distinct profiles depending on the location within the placenta and (2) “semi-composite” 16S profiles using multiple V regions validated by quantitative PCR analysis confirmed that distinct bacterial taxa dominate in different placental niches. Finally, profiles are not altered by mode of delivery. Together these findings suggest that there is niche-specificity to the placental microbiota and placental microbiome studies should consider regional differences, which may affect maternal, fetal, and/or neonatal health and physiology.

## Introduction

The placenta, a transient organ responsible for fetal nutrition, waste disposal, immune tolerance, and maternal-fetal gas exchange, is important for a healthy pregnancy. Traditionally, placental sterility was considered a requirement for a healthy pregnancy because infection and inflammation are strongly associated with adverse pregnancy outcomes (reviewed in ref. 1). However, new studies have revealed that the placenta, much as the rest of the body, harbors microbes, challenging the sterile-womb paradigm. We previously showed, using histological analysis, that intracellular microbes (bacteria) are present in a third of term placental biopsies of the basal plate at the maternal-fetal interface^2^. Additionally, we showed that bacteria were capable homing to and replicating within basal plate explants from term placentas^3^. Likewise, culture-dependent and -independent methods have identified a low-abundance microbiome, the collection of microbial genomes at a particular site, in healthy whole placentas^4^. Similarities between microbial communities in the placental and oral niches have suggested that the oral mucosa is a possible source of the placental microbiome^4, 5^. Additionally, given the phyla-specific similarities of microbes identified in the placenta, amniotic fluid, and infant meconium, it has been proposed that the developing neonate acquires part of its complement of microbiomes from the placenta *in utero* ^6^. Furthermore, commensal bacteria could be isolated from umbilical cord blood^7^. Together, these studies underscore that there are microbial communities present in the placenta and that the fetus at term is not sterile.

The placenta is fetal-derived and develops from the outer layer of the blastocyst-stage embryo. The placenta can be sub-divided into three major components. First, fetal amniotic membranes (FM) encapsulate the fetus and the amniotic cavity. Second, the placental villi (PV) are a network of tree-like projections lined by a multi-nucleated syncytial trophoblast layer and an underlying proliferative cytotrophoblast layer. These cells are in direct contact with maternal blood throughout pregnancy^8^. Third, on the maternal side of the placenta is the basal plate (BP), which is largely responsible for immune-tolerance, defense, and maternal-fetal cross-talk. This compartment harbors several cell-types including maternal immune cells, maternal uterine endothelial cells, and fetal-derived extra-villous trophoblasts (EVTs), which extravasate from the PV to invade the BP and promote spiral artery remodeling to facilitate the flow of blood to the fetal side of the placenta.

A number of studies have demonstrated that the placental compartments have differential barrier capacities. For example, the EVTs in the basal plate are the preferred site of colonization by *L. monocytogenes* and *E. coli* ^3^ and cells in the PV are less susceptible to *L. monocytogenes* ^9^ or Zika virus^10^. This differential susceptibility appears to be due, in part, to the fact that syncytiotrophoblasts of the PV exhibit high basal activity of autophagy, a cellular recycling mechanism that plays an important anti-microbial role, whereas the EVTs cells have low basal autophagic activity^11^.

Given that the different regions of the placenta have distinct functions and barrier capacities, we hypothesized that they would harbour different microbial communities. Here, we used the bacterial 16S variable (V) regions to profile the microbiota membership and abundance of the 16S gene at the three sites: BP, PV, and FM in placentas from normal term pregnancies. We found that the absolute abundance of the 16S ribosomal RNA (rRNA) gene and diversity differed in these three regions. Furthermore, we confirmed the regional specificity of a BP microbial community member at a single species level. Thus, we propose that there is a normal term microbiome, which varies in a location-specific manner.

## Results

### Isolation of site-specific placental samples and microbiome analysis

**Figure 1:**
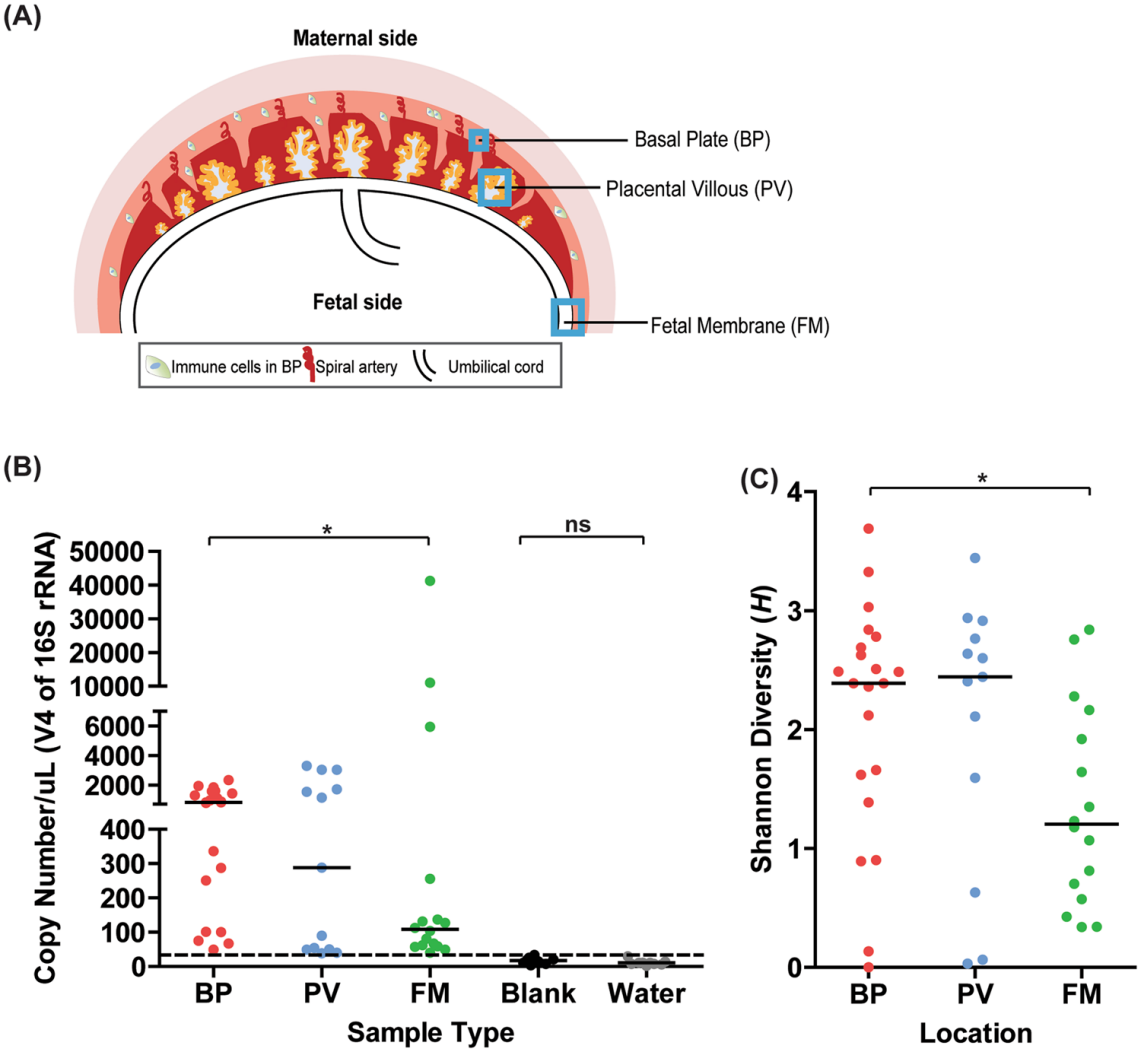
Microbial community profiles in normal term placentas are spatially distinct based on absolute abundance of the 16S gene and alpha-diversity. (**A**) Schematic of the gestational compartment depicting the maternal side (BP) and fetal side (PV and FM). (**B)** Copy numbers/µL of validated V4 samples are plotted per sample type—BP (N = 21), PV (N = 13), FM (N = 16), Blank (N = 8), Water (N = 9). Samples above the dotted threshold (>34 Copies/µL) indicate samples that have met stringency filter set at the maximum copy number/uL of the negative controls (BP vs. FM–P = 0.04; BP vs. PV–0.66; FM vs. PV–P = 0.98; Blanks vs. Water–P = 0.17). **(C**) Shannon diversity score of validated samples grouped by sampling location (FM vs. BP: P = 0.01, PV vs. BP: P = 0.94, PV vs. FM: P = 0.07). Statistical analyses were performed using Mann-Whitney and significant differences are designated with the following annotations: P = *>0.05, *<0.005, *<0.0005.

We obtained placentas from 57 women who delivered at term at the Barnes Jewish Hospital at Washington University^2^. Informed consent for subjects was obtained through the Women and Infants Health Specimen Consortium. Demographic and detailed clinical characteristics of our cohort are presented in Table S1. Our cohort included singleton and twin births. We obtained three samples each from the three sites (BP, PV, and FM) **(**Fig. 1A) and bacterial genomic DNA from the three regions was amplified using bar-coded primers (Table S2) flanking the bacterial ribosomal 16S gene variable regions using the Fluidigm Access Array System and sequenced using the Illumina Miseq platform. Downstream analyses were then performed on the amplicon sequences and Fast-join was used to join paired-end reads^12^.

Recent studies of the placental microbiome have used the 16S regions V1–V3^4^, V3–V4^13^ and V6-8^5^. We attempted to sequence all nine variable regions (V1, V2, V3, V4, V5, V6, V7-8, and V9). However, we found that primers specific to the V7-8 regions did not amplify any sequences. In addition, the V1, V5 and V9 regions were amplified in fewer than half of the samples (Table S3). On the other hand, the V2 and V6 regions generated the greatest number of total reads and were amplified in most samples, but also amplified negative controls (including blanks and water). Analysis was therefore primarily performed using samples amplified with primers flanking the V4 region (F515–R806) as it amplified bacterial DNA in the majority of samples, had a sufficient number of reads for analysis, and had the fewest number of reads detected in the negative controls compared to other variable regions (Table S3; Figs S1–S2).

To quantify community composition, amplicons were clustered into operational taxonomic units (OTUs) using Quantitative Insights into Microbial Ecology (QIIME)^14^ and the Greengenes reference database^15^ and were taxonomically assigned with the UCLUST consensus taxonomy assigner^16^. To minimize risk of including false positive OTUs, we performed a series of filtering steps for analysis. *First*, we removed OTUs observed only once from samples to eliminate potential sequencing artifacts^17^. The average Shannon diversity, which accounts for evenness and richness of the OTUs within a sample^18^, was estimated (following 10 iterations) for each sample in a series of rarefaction depths (step-size = 10)—ranging from 10 to 300 reads. At a sequencing depth of 300 reads, the Shannon diversity estimate of the samples remained stable suggesting that no further diversity could be captured at a higher number of reads (Fig. S3). Thus, we rarefied all samples to a read depth of 300 for all downstream analyses. *Second*, to determine whether the observed OTUs reflected any possible contamination due to purification methods as has been suggested by Lauder *et al*.^19^, we validated the presence of bacteria in samples by absolute quantification of bacterial copy number of the 16S gene per µl of each sample. We used quantitative real-time PCR using primers targeting the V4 region (Table S2) and generated a logarithmic standard curve using the Cq values of a known quantity of *E. coli* gDNA (Uropathogenic *E. coli* or UPEC) that was serially diluted. The total 16S gene copy numbers/µl sample were calculated using the standard curve and Cq values of samples and confirmed to be highly significantly different from negative controls (Fig. S4). All of the V4 positive controls had greater than 14,000 sequencing reads (data not shown). Negative controls (water and reagent test blanks) occasionally exhibited a signal at 34 copies/µL and therefore we excluded any sample at this threshold and below from downstream alpha and beta-diversity analysis. *Finally*, negative control data (water, N = 5; extraction and purification blanks, N = 8) and positive controls (*E. coli* DNA, N = 8) revealed an occasional OTU in the blanks and water, but most did not fit within our filtering criteria.

### The microbial community profile in normal term placentas exhibits spatial variation

We determined whether we could identify differences in the microbial profiles in the validated BP, PV, and FM samples (Fig. 1A). We first compared the 16S copy number in the location-specific samples and found that the median copy number was significantly higher in the BP, at the maternal-fetal interface, than in the FM, which faces the amniotic cavity, whereas the BP and PV samples had similar median 16S copy numbers (Fig. 1B). Next, we used Shannon Diversity Index (*H*) to determine the alpha diversity (diversity within samples) between the BP, PV, and FM groups. We found that the median Shannon diversity was lower in the FM than in the BP, but the BP and the PV had similar levels of diversity (Fig. 1C). Together, these findings demonstrate that the abundance and diversity of microbes differ by sampling location in the placenta.

**Figure 2:**
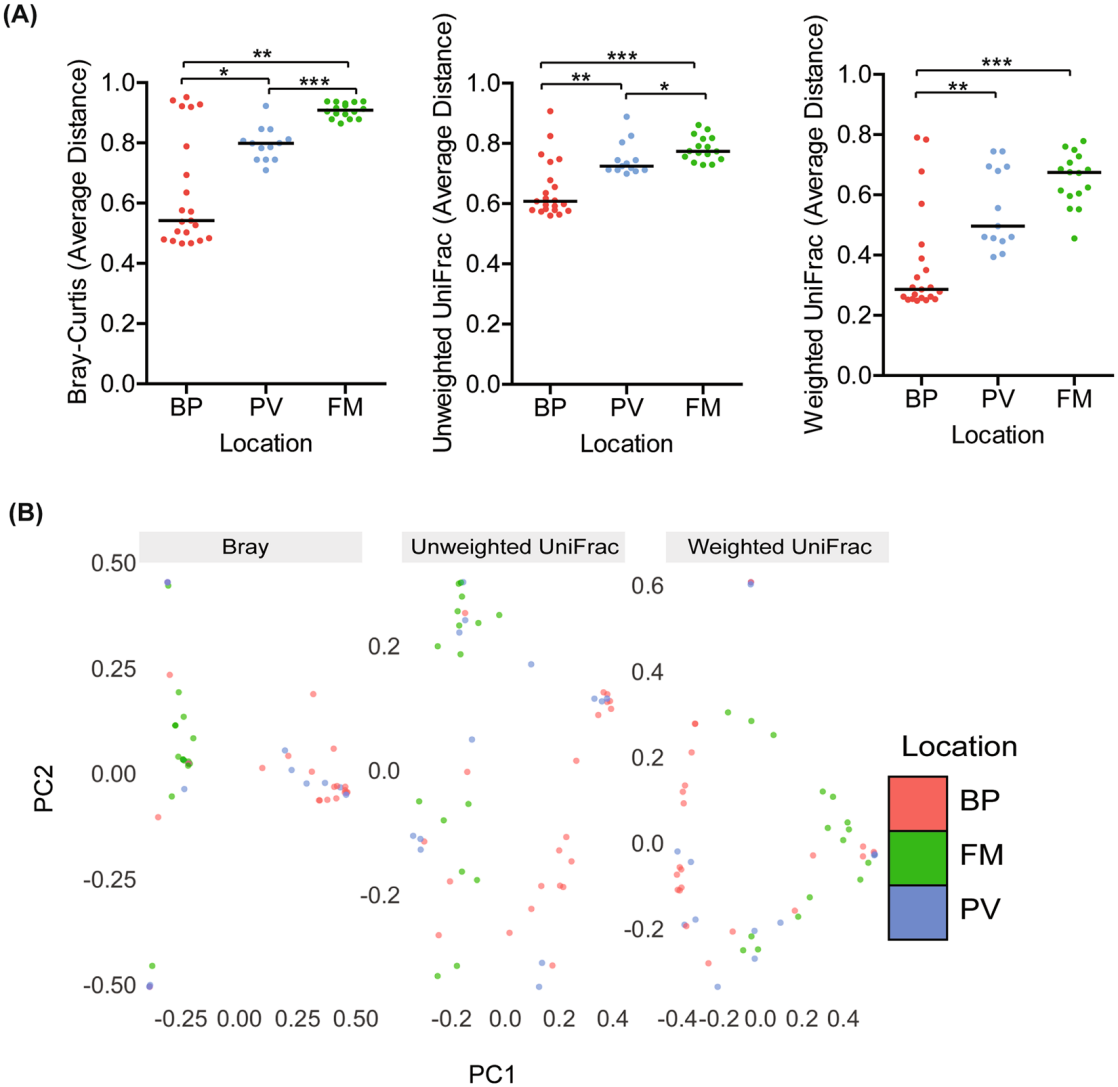
Beta-diversity analysis illustrates greater similarity of microbial profiles within the basal plate samples than in samples from fetal membranes. (**A**) QIIME generated Bray-Curtis, unweighted Unifrac, and weighted UniFrac of average distances of validated samples grouped by sampling location. By all metrics, samples within the basal plate were significantly more similar to one another compared to samples within the placental villous or fetal membrane based on the median distance within each group (BP vs. PV–Bray-Curtis: P = 0.018; unweighted UniFrac: P = 0.0029; weighted UniFrac: P = 0.0011; BP vs. FM–Bray-Curtis: P = 0.0014; unweighted UniFrac: P = <0.0001; weighted UniFrac: P = 0.0002). Samples in the placental villous were significantly more similar to each other than samples within the fetal membrane (PV vs FM:–Bray-Curtis: P = <0.0001; unweighted UniFrac: P = 0.015; weighted UniFrac: P = 0.083). (**B**) Multidimensional scaling (MDS) plot of samples within the BP, PV, and FM based on the Bray-Curtis, unweighted UniFrac, and weighted UniFrac distance metrics (PERMANOVA: Bray: P = 0.001, weighted UniFrac: P = 0.001, weighted UniFrac: P = 0.001). Statistical analyses were performed using Mann-Whitney and PERMANOVA, and significant differences are designated with the following annotations: ﻿﻿P =﻿ *<0.05, **<0.005, ***<0.0005.

Next, we employed beta-diversity analysis to evaluate whether there was spatial variation between the samples from each placental location. QIIME-based beta-analysis was used to assess pairwise dissimilarity between samples within the same location. To measure dissimilarity we used Bray-Curtis method (which considers the relative abundance of an OTU between samples), unweighted UniFrac (which considers OTU similarities between the samples), and weighted UniFrac (which considers the types of OTUs present and the relative abundance of OTUs between samples). For each diversity metric, we determined the average dissimilarity per sample. Samples were grouped by the location from which biopsies were taken. Samples with averages closer to 1 indicates maximum dissimilarity and samples close to 0 indicates that samples are completely similar based on the metric used. By all metrics, assessment of median distances within each group revealed that samples from the BP were significantly more similar to one another compared to similarities between samples within the PV or FM (Fig. 2A). Further, by Bray-Curtis and Unweighted UniFrac, samples within the PV were significantly more similar to one another than samples in the FM).

Multidimensional scaling (MDS) analysis or supervised clustering analysis was also applied to the validated V4 dataset. Principal components analysis was used to model the variation between samples^20^ based on location and the data revealed that the centroids or average center, differed significantly between all three locations (Fig. 2B **;** Fig. S5). These findings suggest that there is more similarity within the BP or PV samples than similarity of samples within the FM.

### Mode of delivery does not substantially affect the microbial profiles

**Figure 3:**
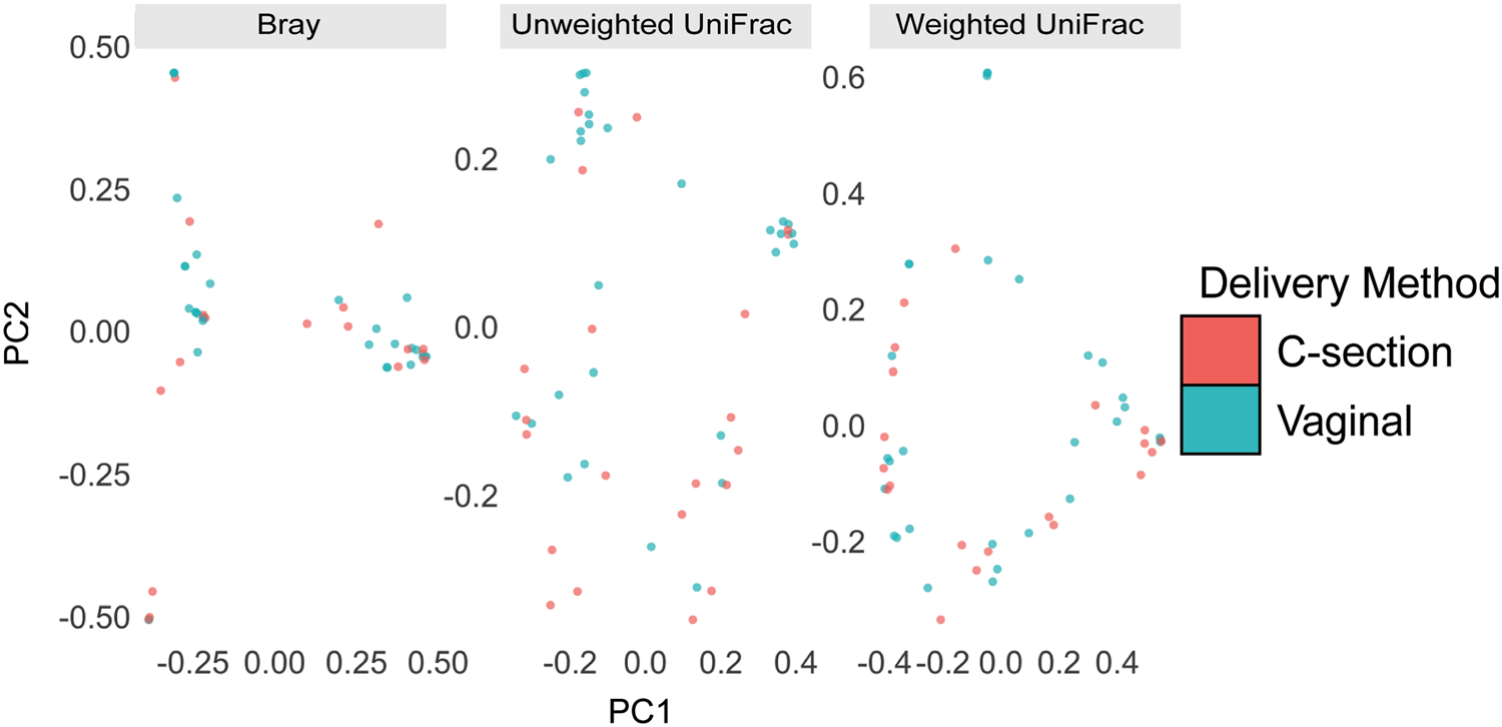
Mode of delivery does not alter the microbial profiles. Multidimensional scaling (MDS) plot of validated samples by delivery method based on the Bray-Curtis, unweighted UniFrac, and weighted UniFrac distance metrics. The centroid of average distances per delivery method was not significant (PERMANOVA: Bray-Curtis: P = 0.49, Unweighted UniFrac: P = 0.060. Weighted Unifrac: P = 0.38). Statistical analyses were performed using the Mann-Whitney test and PERMANOVA.

Given the relatively low abundance of the placental microbiome, there is a possibility that there may be contamination from the vaginal tract upon delivery. We reasoned that if the microbiota we detected was due to contamination during vaginal delivery, then we should detect differences between microbiota of placentas delivered vaginally and those delivered by cesarean section. Thus, we analyzed 21 samples, representing all placental regions, from women that delivered by Cesarean section and 29 samples, representing all placental regions, from women that delivered vaginally. To assess the impact of mode of delivery on beta-diversity, we performed supervised (MDS) clustering of the pairwise beta-diversities for each sample to visually represent the variations or lack thereof between samples, with each axis representing the 1^st^ and 2^nd^ principal coordinates (Fig. 3). The centroid was determined for each delivery method given the pairwise beta diversities for each sample. The differences in centroids were evaluated for statistical significance. Our findings further confirmed that the delivery method did not significantly affect the microbial profiles. This data suggests that the placental microbiota profile is not altered by mode of delivery.

### Microbial taxa exhibit geographical differences

**Figure 4:**
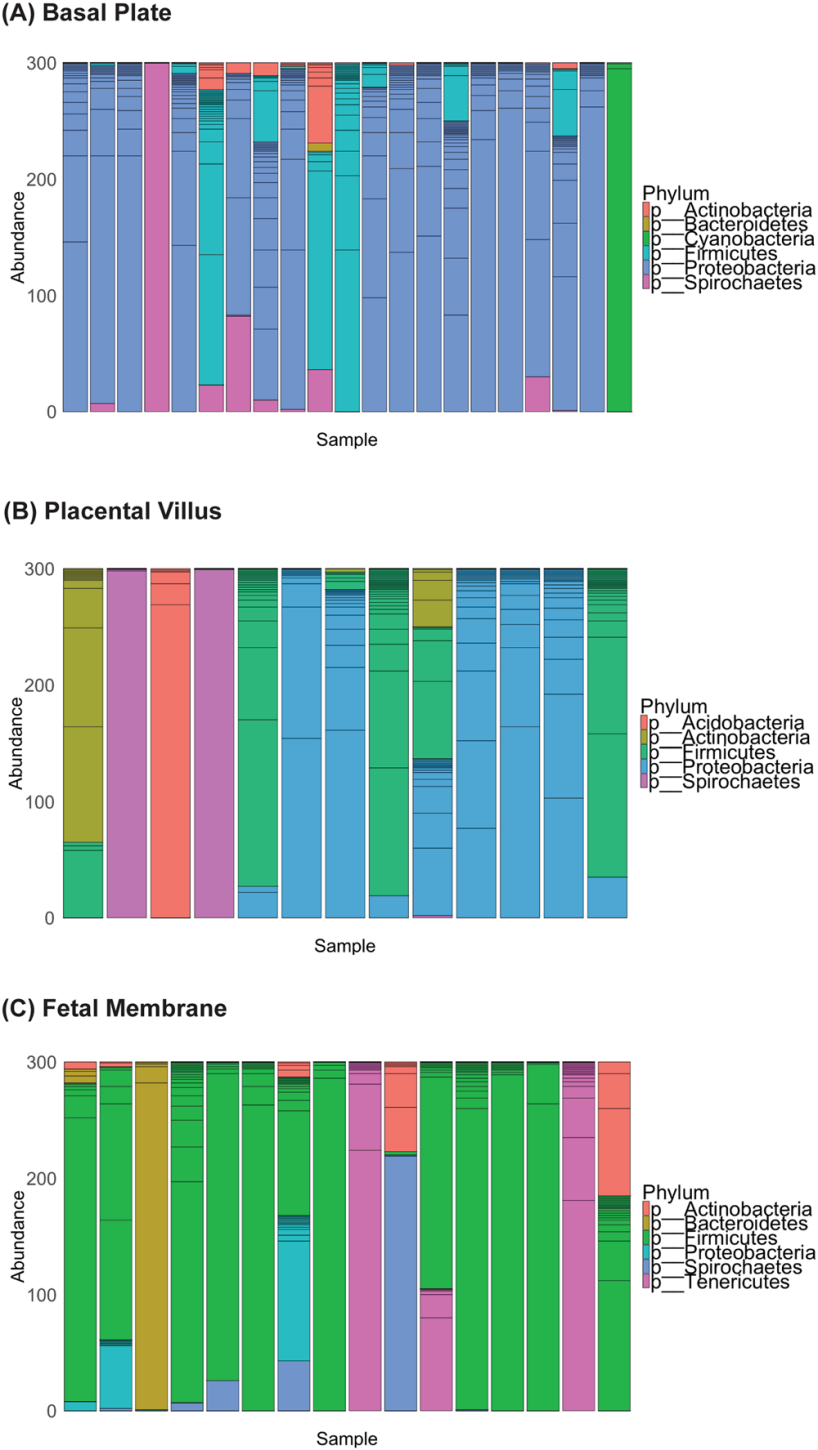
Spatial variation of microbial taxa in the placenta. Patterns of bacterial phyla (colors) relative abundance at a given placental site—(**A**) Basal Plate, (**B**) Placental Villus (**C**) Fetal Membrane. Each bar represents a different validated sample. Unique taxa within a specific phyla are designated by additional lines within each bar.

We next assessed taxonomic similarities between each placental location by comparing the bacterial phyla in the BP, PV, and FM samples. We found six bacterial phyla in the BP samples (*Proteobacteria, Actinobacteria*, *Bacteriodetes*, *Cyanobacteria*, *Firmicutes*, and *Spirochaetes)* and these phyla were represented by 185 unique taxa. The PV samples harbored five phyla (*Acidobacteria, Actinobacteria, Firmicutes, Proteobacteria*, and *Spirochaetes*) and were represented by 144 unique taxa. Six phyla in the FM samples were identified (*Actinobacteria*, *Bacteriodetes*, *Firmicutes*, *Proteobacteria*, *Spirochaetes*, and *Tenericutes*,) and were represented by 168 unique taxa **(**Fig. 4A–C **)**.

**Figure 5:**
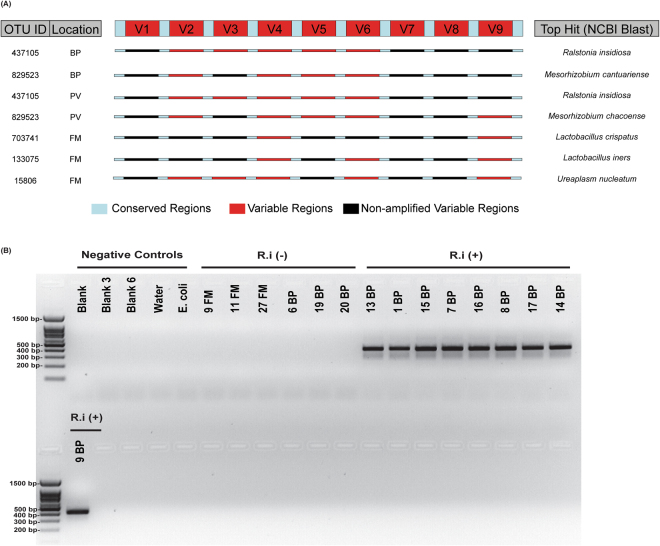
Species-specific analyses using multiple variable regions and species-specific primers. (**A**) (Left Panel) Summary of the most prevalent OTU IDs and the selected representative samples (chosen based on having the highest number of detectable variable regions and were not present in the V4 negative controls for each location/OTU ID. (Top Center) Schematic of the 16S rRNA gene, with conserved regions (light blue) and variable regions (red). (Center) Schematic of the 16S rRNA gene for each representative sample, where red indicates amplified variable regions and black indicates regions that were not amplified for that sample. (Right panel) Top BLAST hits for each OTU ID, based on the “semi-composite” sequence generated from amplified variable regions of representative samples. **(B)** Amplification of qPCR product for *R.i* (403 bp) in BP samples, which were positive by 16S rRNA sequencing. The full-length gel shows molecular sizes of markers of the 100 base pair ladder and negative controls, including blanks, water, *E. coli* plus BP and FM samples that were not positive for *R.i* according to sequencing analyses. Samples were from the same qPCR analysis and run on the gel together.

Next, we sought to determine the most highly prevalent and unique microbial species in a given niche. We identified OTUs with the highest prevalence in each location and excluded those OTUs that were also detected in the V4 negative controls. Using the Basic Local Alignment Search Tool (BLAST), sequences from the detectable variable regions in a given sample were queried together against 16S rRNA databases and the top hits from the BLAST search identified (Fig. 5A)^21^. Our data indicate that the species with highest prevalence in the BP as well as PV included *Ralstonia insidiosa* and *Mesorhizobium spp*. On the other hand, the most prevalent species in the fetal membranes were *Lactobacillus crispatus*, *Lactobacillus iners*, and *Ureaplasma nucleatum*.

*Ralstonia insidiosa* (*R. i*.) from the Burkholderiales order was found in 76% of the validated BP samples. We performed quantitative PCR analysis on *R. i*. and validated that *R. i*. could be amplified using specific primers to this species only in BP samples, which had *R.i* OTUs and not in fetal membrane samples where it was not detectable (Figs 5B and S6). Together, our findings support the geographical niche specificity to microbial communities in the placenta with taxa associated with the fetal membranes most closely related to those typically found in the vaginal niche and distinct from the taxa found in the basal plate and placental villous regions of the placenta.

## Discussion

The long-held paradigm of the sterile womb has been challenged, and the presence of a microbiome in pregnancy-associated organs has begun to be investigated in greater detail^4, 6, 22, 23^. Given that the placenta serves a number of site-specific functions, we profiled the microbes in specific locations on the maternal and fetal sides of the gestational compartment. Here, we demonstrate that there are distinct differences between the fetal membrane and basal plate regions based on 16S abundance as well as bacterial diversity at the phyla and species levels.

Studies employing the 16S sequencing method to identify microbes present in the placenta during gestation or to prove otherwise have focused on specific variable regions^24^. A strength of our study is that we used all variable regions of the 16S gene and found that the choice of variable region could alter the generation or interpretation of data. For example, we did not note any sequences using primers flanking the V7-V8 regions. Further, we detected a greater number of reads in blanks and water samples using other variable regions such as V1/V2/V6 relative to V4. Thus, studies on low abundance microbiomes such as those found in the placenta, fetal and neonatal gut, neonatal lung or in the urinary tract should consider sequencing microbiota flanking primers to multiple variable regions of the 16S rRNA gene to identify the region that most reliably distinguishes signal from noise and permits rigorous data interpretation.

Furthermore, as has been suggested by a recent study^19^, studies of pregnancy microbiome or others with low microbial biomass samples in general should also consider absolute abundance measures such as qPCR of total 16S rRNA gene copies to avoid false positive signals and validate the findings from sequence analysis. Finally, we took a number of measures to ensure that we did not simply sequence contaminants as may occur for example, during vaginal delivery or handling of the samples, or in reagents. Our findings indicate that the mode of delivery does not have any significant effect on the microbiota identified in the placental regions, underscoring that they are present before birth and not acquired in the birthing process. In contrast, mode of delivery may affect microbial communities in the peri-partum period in samples such as neonatal feces^25–27^.

Previous studies on the placental microbiota have been on biopsies of whole placentas. A significant strength of our study is investigation of the microbial communities in various niches within the placenta, given that the placenta is a complex structure comprising cells from maternal and fetal (and thus also genetically paternal) compartments and thus likely to exhibit regional variations. Furthermore, a number of studies from our group and others have identified that there are differential susceptibilities to microbial colonization in trophoblasts from the basal plate or the placental villi^3, 11^. Indeed, our 16S rRNA sequencing studies demonstrated distinct differences in the microbial profiles based on location. Interestingly, but perhaps not surprisingly, the differences are greatest between the basal plate, which comprises more maternal elements, and the fetal membranes, which are of fetal origin. Furthermore, there are few to no differences between the basal plate and placental villi, and there are a number of sample to sample variations in the villous samples, suggesting that the villi may not harbor any true resident microbes and may reflect transient microbes in the maternal blood that bathe the villi. This finding, if true, would be consistent with our recently published work showing that the villous trophoblasts comprise a formidable barrier to infection with high levels of basal autophagy^11, 28^. Autophagy, a cellular degradation pathway, may effectively degrade microbes, commensal or pathogenic such that any reads we observe may not be representative of live microbes. This remains to be elucidated further.

Another strength of our study is that species-specific analysis underscore the geographical influences of the placental niche. Overall, we found that the BP samples were dominated by *Proteobacteria*, whereas the FM samples were dominated by *Firmicutes* while the PV samples showed no consistent dominating phyla. Aagaard *et al*. reported a preponderance of *Proteobacteria* in whole placental tissues, which may suggest that may suggest that their sampling represented more of the microbial phyla in the maternal fetal interface^4^. Similar to their findings, we noted Gamma-proteobacteria in the basal plate; however, we did not identify any *E. coli* species in the placenta. A limitation of our study is that our findings do not provide us sufficient evidence to discuss the significance or origin of the microbial phyla observed at any given placental niche. However, we can speculate that given the basal plate is in direct contact with the uterine epithelium, it may be a site from which the microbes in the basal plate originate. *Bacteroidetes*, which are prevalent in the basal plate have been found in the non-pregnant uterus^29^.

Interestingly, our species-specific analysis revealed the presence of *Ralstonia insidiosa* in the basal plate samples and not in fetal membrane samples. The *Ralstonia* genera comprise gram negative bacterial species such as *Ralstonia insidiosa* and are nonfermentative, aerobic bacilli that can form biofilms and survive in low nutrient conditions^30^. They have been associated with the microbiomes in breast milk^31^. We have previously shown that the basal plate harbors intracellular microbes within extravillous trophoblasts^2^. We further identified that in some cases, the intracellular microbes are present as clusters of bacilli suggestive of small biofilms^2, 3, 32^. Thus, we can speculate that species such as *Ralstonia insidiosa* could potentially exist intracellularly in trophoblasts at the maternal-fetal interface and may present bacterial antigens to the maternal immune cells in the basal plate. Recently, a study in mice has demonstrated that maternal microbiota in pregnancy can contribute to neonatal immune system development^33^. Our findings also show that *Lactobacilli*, which have previously been detected in the human placenta using species-specific PCR analysis^34^, dominate in the fetal membranes. Species-specific analysis using multiple variable regions confirmed the presence of *Lactobacillus crispatus* and *Lactobacillus iners* in the fetal membranes. Notably, these species are prevalent in the intestinal and vaginal flora, which might suggest that the fetal membranes are exposed to microbial species ascending from the vagina.

Although our studies have revealed tissue-specific profiles in the placental microbiome, this initial characterization is only the first step in determining whether microbes play a biological role during pregnancy. Many questions remain including (1) how does one distinguish between infectious pathogens and commensal microbes in the placenta? (2) whence from do these microbes originate? (3) what is the role, if any, for these microbes? Future investigations into this new field of the pregnancy microbiome may lead to new understanding of development of fetal and neonatal immunity, normal parturition and adverse pregnancy outcomes.

## Materials and Methods

### Study Design and Tissue Collection

This cross-sectional study was designed as described in^2^ and was approved by the Institutional Review Board of Washington University School of Medicine St. Louis, MO (IRB ID 201012734). Informed consent was obtained from all subjects used in this study. All methods were performed in accordance with the guidelines and regulations of Washington University School of Medicine. Specimens were derived from term subjects, since we were primarily interested in spatial diversity and abundance of microbes under normal conditions. Our cohort included 57 term women, 34 who underwent Cesarean section and 23 of who delivered vaginally. Detailed characteristics of term cases are presented in Table S1. Placentas from 57 term mothers were biopsied ≤12 hours after delivery. Trained research assistants used sterile technique to harvest 5-8 mm samples from the placental villous, fetal membrane, and the basal plate, as described^2^. Samples were placed in sterile cryovials, snap frozen in liquid nitrogen, and stored at −80 °C until DNA extraction.

### Extraction and Purification of bacterial genomic DNA from term placental tissue

The basal plate, placental villus, and fetal membrane of the placenta were processed and genomic DNA was isolated from each location. Extraction and purification of samples were performed using autoclaved tools. DNA extraction and purification was performed in a laminar-flow hood that wiped with bleach and ethanol. As the human gDNA was the predominant component relative to bacterial DNA, bacterial gDNA was further purified using the QiaQuick PCR Purification Kit (QIAGEN). DNA extraction of samples was performed according to the DNeasy Blood and Tissue Kit (QIAGEN) protocol. Briefly, frozen tissue samples were suspended in tissue lysis butter ALT. Tissues were homogenized using sterilized magnetic beads at a frequency (1/s) of 30 using TissueLyser II (QIAGEN). Homogenized samples were centrifuged using the Centrifuge 5417 R (Eppendorf). 20 μL of proteinase K was added to each sample and incubated in the 56 °C shaker for 3 hours. gDNA was eluted into 200 μL of molecular biology grade water (CORNING). Negative control blanks, which included ‘no tissue’, and molecular biology grade water, were subject to all steps of the DNA extraction and purification procedure including proteinase K and homogenization steps. Samples were quantified using NanoDrop Spectrophotometer (Thermo Scientific). Approximately 10 ng/μL of each purified (QIAquick Purification Kit, QIAGEN) DNA sample, blank, and molecular biology grade water were loaded into autoclaved 96-well PCR plates (BIO-RAD) and sealed with Microseal B Adhesive Seals (BIO-RAD). Samples were sent to the Genome Technology Access Center (GTAC) for 16S PCR amplification, sequencing, and sequencing data analysis.

### Amplicon generation and Sequencing of Bacterial 16S rRNA Genes

Purified bacterial gDNA was then used for amplicon generation and next generation sequencing on the MiSeq platform using the bacterial ribosomal 16S gene primers (Table S1). Variable regions targeting V1–V9 were used. The Fluidigm Access Array System was used to construct 14 PCR amplicons, representing all 9 16S variable regions. 1X High Fidelity FastStart Reaction Buffer without MgCl_2_, 4.5 nM MgCl_2_, 5% DMSO, 200 μM PCR Grade Nucleotide Mix, 0.05 U/μL 5 U/μL FastStart High Fidelity Enzyme Blend, 1X Access Array Loading Reagent (Fluidigm), 1 μl of DNA at a concentration of 5 ng/μl, and water were put into each sample inlet. To add primers to the assay inlets, 200 nM forward and reverse primers were combined with the 1X Access Loading Reagent. PCR amplification of harvested inlet samples was performed on the Fluidigm Biomark. Each sample was then harvested and indexed using unique 10 base pair sequences with 14 rounds of PCR to incorporate each index sequence. All samples were pooled into 48 sample libraries and cleaned using bead purification. Samples were loaded and sequenced using the Illumina MiSeq platform. Primers used for 16S amplicon generation are shown in Table S2.

### Sequencing Filtering, OTU Clustering, and alignment

Downstream analyses were performed on the PCR amplicons. Fast-join was used to join paired-end reads^12^. Sequences that corresponded to a particular amplicon were identified using their matching/corresponding primer sequences. 16S rRNA sequences were uploaded to the NCBI Sequence Read Archive (BioProject PRJNA395716). The Quantitative Insights to Microbial Ecology (QIIME) pipeline version 1.9.0 was used for read analysis^14^. In the QIIME analysis, each amplicon and sample pair was defined as a separate sample. Open-reference operational taxonomic units (OTU) were called using the Greengenes May 2013 release as the reference database^15^. Reads were clustered into OTUs by QIIME using UCLUST^16^ at a threshold of 97% similarity. Representative sequences for each OTU were classified taxonomically with the UCLUST consensus taxonomy assigner in QIIME using a sequence similarity of 0.9. To ensure that the de-novo OTUs aligned to 16S sequences and were not randomly constructed, we opted to use the no_pynast_failures file and removed OTUs that did not align with the pynast to the Greengenes core alignment.

### 16S rRNA Quantitative real-time qPCR

We performed qPCR, targeting the conserved V4 region of the 16S gene (primers F515: 5′ GTGCCAGCMGCCGCGGTAA 3′ and R806: 5′ GGACTACHVGGGTWTCTAAT 3′) in samples that had been filtered by removing OTUs observed once and rarefied to 300 OTUs. Purified extraction and purification blanks (that had been sequenced) and fresh molecular biology grade water (CORNING) were used as negative controls.

To calculate the 16S copy numbers per sample, a standard curve was generated using *E. coli* DNA. To do this, a laboratory stock of *E. coli* was streaked onto Luria Bertani (LB) agar plates and grown at 37 °C overnight. A colony was selected and inoculated into LB broth, grown overnight at 37 °C, centrifuged and the pellet re-suspended in 10 mls of PBS. 1 mL was used for the extraction as described﻿ above. *E. coli* 16S rRNA gene exists as 7 copies^35^ in each cell with a genome size of 5.18 Mb^36^. Using this information and given the concentration of the *E. coli* DNA stock, we calculated the quantity of 16S copies per uL of this stock. To generate the standard curve, a series of five 10-fold dilutions was generated from this initial stock.

Each quantitative polymerase chain reaction (qpcr) included 6 standards and the selected placental samples run in triplicate. Each 20 uL qPCR reaction consisted of 0.5 μL of 1X forward primer (71.4 ng/μL) and R (61.1 ng/μL) primer, 10 μL of Sso Advanced Universal SYBR (BIO-RAD), 5 μL of molecular biology grade water, and 1.0 of DNA template (at ~10 ng/μL) diluted in 3 μL of molecular biology grade water. Samples were loaded on into Hard-Shell 96-Well PCR Plates (BIO-RAD) and sealed with Microseal B Adhesive Sealers (BIO-RAD). These steps were performed in a laminar flow hood.

Quantitative PCR was performed using the C1000 Touch ThermoCycler (BIORAD) and the software and CFX Manager Software Version 3.1 (BIO-RAD). Cycling conditions were as follows: 94 °C for 10 minutes, and 40 cycles of 95 °C for 15 seconds, 60 °C for 1 minute. We prepared a logarithmic standard curve based on the calculated copy number per standard and their corresponding average Cq values. Based equation of the standard curve, we calculated the copy number of each sample using its average Cq^37^.

### *Ralstonia insidiosa-*specific qPCR

We performed qPCR using *R.i*-specific primers designed previously (Rp-F1 5′ ATGATCTAGCTTGCTAGATTGAT 3′ and R38R1 5′ CACACCTAATATTAGTAAGTGCG 3′)^38^ (Integrated DNA technologies) to confirm *R.i* in samples positive for *R.i* (BP, N = 9) and negative in samples that did not detect this particular taxa during sequencing analysis (FM, N = 3 FM and BP, N = 3). Blanks (N = 3), water (N = 1), and *E. coli* (N = 1) were used as additional negative controls. qPCR cycling conditions were used as described previously. qPCR product was subsequently run on 2% agarose gel comprising of 2.0 g of agarose Standard Agarose (LAMDA BIOTECH), 100 mls of TAE buffer, and 1 μL of ethidium bromide 1% solution (Fisher Scientific). 2 μL of 5x GelPilot Loading Dye (QIAGEN) was loaded into 20 μL qPCR product and 10 μL of each sample was loaded into each well. Samples were run using the EC-105 Compact Power Supply (E-C apparatus Corporation) with a 100 base-pair DNA ladder **(**LAMDA BIOTECH**)** at approximately 100 Volts for 30 minutes. The agarose gel was photographed under ultraviolet light using AlphaImager 2200 (Alpha Innotech) using the software Alpha Ease FC Version 3.2.1.

### α-Diversity Analysis

To compare the α-diversity (diversity within samples) the basal plate, placental villous, and fetal membranes, the QIIME commands alpha_diversity,py and collate_alpha.py were performed to generate the estimated Shannon diversity indices for validated samples. Consistent with sample validation pipeline, OTUs observed once were not considered in this analysis and samples were analyzed at a read depth of 300. At this depth, the estimated Shannon diversity (after 10 iterations) for each sample was averaged. Samples were grouped based on the site from which they were biopsied. The median Shannon diversity per site was statistically compared between sites.

Using the QIIME command, single_rarefaction.py, we rarefied the OTU biom files of validated samples to a read depth of 300. This biom file and rep_set.tre, a phylogenetic tree of OTUs within samples generated by QIIME, were imported in to *R* and the package “Phyloseq” was used to visualize the relative abundance of bacterial phyla in each sample, based on the taxonomic classification of 16S sequences called using Greengenes. Samples were grouped based on the site from which they were biopsied.

### β-Diversity Analysis: Average Pairwise-Distances and Multidimensional Scaling Analysis

Using the phylogenetic tree generated by QIIME, rep_set.tre, QIIME outputs for beta_diversity.py and make_distance_plots.py, we generated a pairwise dissimilarity matrix table of validated samples grouped by site^38, 39^. These dissimilarity matrices were generated on the following community distance metrics: unweighted UniFrac, weighted UniFrac, and Bray Curtis. Pairwise distance values using unweighted UniFrac were calculated by comparing the fraction of total branch lengths that are unshared between OTUs in two samples^38^. To generate weighted UniFrac distance measurements, the phylogenetic profile and relative abundance of OTUs were compared between pairs of samples^38^. Finally, Bray-Curtis distance values involve comparing the relative abundance of a given OTU between pairs of samples^40^. In order to generate a site-specific ‘average distance value’, we averaged together the pairwise distances that corresponded to a particular sample and the other samples within the same sampling location. We then statistically compared the median ‘average distance value’ between sites. The biom file of validated samples and rep_set.tre (or “Phyloseq”-generated random_tree) were used to perform multidimensional scaling analysis based on the V4 dataset using R “Phyloseq” (Figs 2B, S5). In this plot, principal components 1 (PC1) and 2 (PC2) for the dataset were determined based on the component that best explained the variation between samples^20^.

### Species Identification of Prevalent OTU IDs using Multiple Variable Regions

Top OTU IDs were selected based on (1) the prevalence of OTU across samples within a group (BP, PV, or FM), (2) the highest total number of reads for that OTU across samples within each group and (3) were not detected in the negative controls. The reference sequence was identified for the selected OTUs. For each validated sample, the amplicon nucleotide sequence associated with the detectable variable regions were identified. For each location and OTU ID, samples with the highest number of detectable variable regions were selected. For these samples, each amplicon was queried against its associated OTU reference to confirm sequence identities at ~97 using the NCBI Basic Local Alignment Search Tool (BLAST)^21^. For the selected samples, the sequences of the detectable variable regions were input together into BLAST and queried against the 16S Ribosomal RNA Sequences (Bacteria and Achaea) databases. The top BLAST hit was documented.

### Bioinformatics and Statistical Analyses

To compare the median Shannon diversity or copy number between two sample groups, we used the non-parametric Wilcoxon-Mann-Whitney test because our sample sizes for each group did not meet the normality assumption. We followed the same procedure for the beta-diversity analysis to compare the average pairwise distances between locations and to compare 16S copy number/μL between groups.

Two-dimensional MDS plots and sample-specific relative abundance graphics were generated using “R” version 3.3.1. The following “R” packages were used: phyloseq (v. 1.16.2 and 1.19.1), ggplot2 (v. v. 2.1.0 and 2.2.1), vegan (v. 2.4.0 and 2.4.3), plyr (1.8.4), and devtools(1.12.0), cluster (v. 2.0.4), igraph (1.0.1), gridExtra (v. 2.2.1), and ape (v. 3.5)^20, 41^. PERMANOVA was completed to test the null hypothesis that all three locations have the same centroid, or average center, given the pairwise beta diversities. The same analysis was performed to test the null hypothesis that delivery method affected the centroid locations. PERMANOVA was completed for each of the three beta diversity measures — Bray-curtis, unweighted-UniFrac, and weighted Unifrac.

## Electronic supplementary material

Supplemental material
