# Supplementary material for: Microbial communities in placentas from term normal pregnancy exhibit spatially variable profiles
Source: Sci Rep. 2017 Sep 11;7:11200. doi: 10.1038/s41598-017-11514-4 (PMC5593928; doi:10.1038/s41598-017-11514-4)

Microbial communities in placentas from term normal pregnancy exhibit spatially variable profiles

Lindsay A. Parnell[1] Catherine M. Briggs[1*], Bin Cao[1*], Omar Delannoy-Bruno[1], Andrew E. Schrieffer[2], Indira U. Mysorekar[1,3$]

Supplementary Table Legends

Table S1: **Clinical and Demographic Features of Human Subjects**

Table S2: **Sequences of primer pairs used to generate 16S amplicons (V1 – V9)**

Table S3: **Read and Sample Summary of V1-V9 datasets with detectable OTUs**

Clinical characteristics	Mean ± SD
Age (yrs)	28.86 ± 0.67
BMI at first pregnancy visit	29.35 ± 1.21
BMI at delivery	33.90 ± 1.06
Infant weight (g)	3187.39 ± 88.16
Gestation Age (days)	273.19 ± 1.25

			Percent
Race	Asian/ Pacific Islander	2	3.51%
	Black	19	33.33%
	Native American	1	1.75%
	White	35	61.40%
Gestation Number	1	50	87.72%
	2	7	12.28%
	3	0	0.00%
Fetal Sex	Male	25	43.86%
	Female	32	56.14%

	Number (yes)	Percent (yes)
Vaginal Delivery	34	59.65%
Cesarean Section	23	40.35
Multiple Gestation	7	12.28%

Table S1: **Clinical and Demographic Features of Human Subjects**

Amplicon Size	Variable Region	Primer Name	Fluidigm Forward Adapter	Forward Sequence	Primer Name	Fluidigm Reverse Adapter	Reverse Sequence	Miseq 2x150 F/R overlap	Strand bias F,R	PMID	Forward Pubmed historic name	Reverse Pubmed historic name
113	V1	V1	ACACTGACGACATGGTTCTACA	AGAGTTTGATCMTGGCTCAG	V1	TACGGTAGCAGAGACTTGGTCT	TTACTCACCCGTICGCCRCT	all	0	18047683		
261	V2	V2	ACACTGACGACATGGTTCTACA	AGYGGCGIACGGGTGAGTAA	V2	TACGGTAGCAGAGACTTGGTCT	CYIACTGCTGCCTCCCGTAG	39	111,111	18047683		
170	V3	V3_2	ACACTGACGACATGGTTCTACA	CCTACGGGAGGCAGCAG	V3_2	TACGGTAGCAGAGACTTGGTCT	GTATTACCGCGGCTGCTGG	130	20,20	22853944, 21460107	F341	R518
250	V4	V4	ACACTGACGACATGGTTCTACA	GTGCCAGCMGCCGCGGTAA	V4	TACGGTAGCAGAGACTTGGTCT	GGACTACHVGGGTWTCTAAT	50	100, 100	22179717, 20534432	F515	R806
100	V5	V5_2	ACACTGACGACATGGTTCTACA	AGGATTAGATACCCT	V5_2	TACGGTAGCAGAGACTTGGTCT	CRTACTHCHCAGGYG	all	0	22853944	784F	880R
167	V6	V6_1	ACACTGACGACATGGTTCTACA	AAACTCAAAKGAATTGACGG	V6_1	TACGGTAGCAGAGACTTGGTCT	ACGAGCTGACGACARCCATG	133	17,17	18047683		
300	V7-V8	V7-V8	ACACTGACGACATGGTTCTACA	GYAACGAGCGCAACCC	V7-V8	TACGGTAGCAGAGACTTGGTCT	GACGGGCGGTGWGTRC	0	all	20880993	1099	1407
116	V9	V9	ACACTGACGACATGGTTCTACA	GTACACACCGCCCGT	V9	TACGGTAGCAGAGACTTGGTCT	TACCTTGTTACGACTT	all	0	18047683		

Table S2. **Sequences of primer pairs used to generate 16S amplicons (V1 – V9)**

Variable Region	Mean read number	St. error read number	Total read number	Max read number	Number of Samples
V1	1224.64	218.33	106544	10627	87
V2	4622.7	374.3	855200	21637	185
V3	1088.16	192.12	178458	18400	164
V4	2160.91	277.61	345746	23972	160
V5	1492.38	273.55	141776	17737	95
V6	13733.23	988.06	2609314	58301	190
V9	1201.23	201.09	108111	11342	90

Table S3: **Read and Sample Summary of V1-V9 datasets with detectable OTUs**

Supplementary Figure Legends

Fig. S1: **Detected microbial taxa within control samples.** Total number of OTUs (y-axis) detected within each phyla (x-axis) by variable region (row) for a given control (column). The dots within the bars represent unique samples within respective control group (E. coli, n = 8; Blank, n = 8; Water, n = 5). The height of each bar represents total OTUs detected in each control. Each colored bar represents relative abundance of a given phylum. The horizontal lines within each phylum represent a unique OTU.

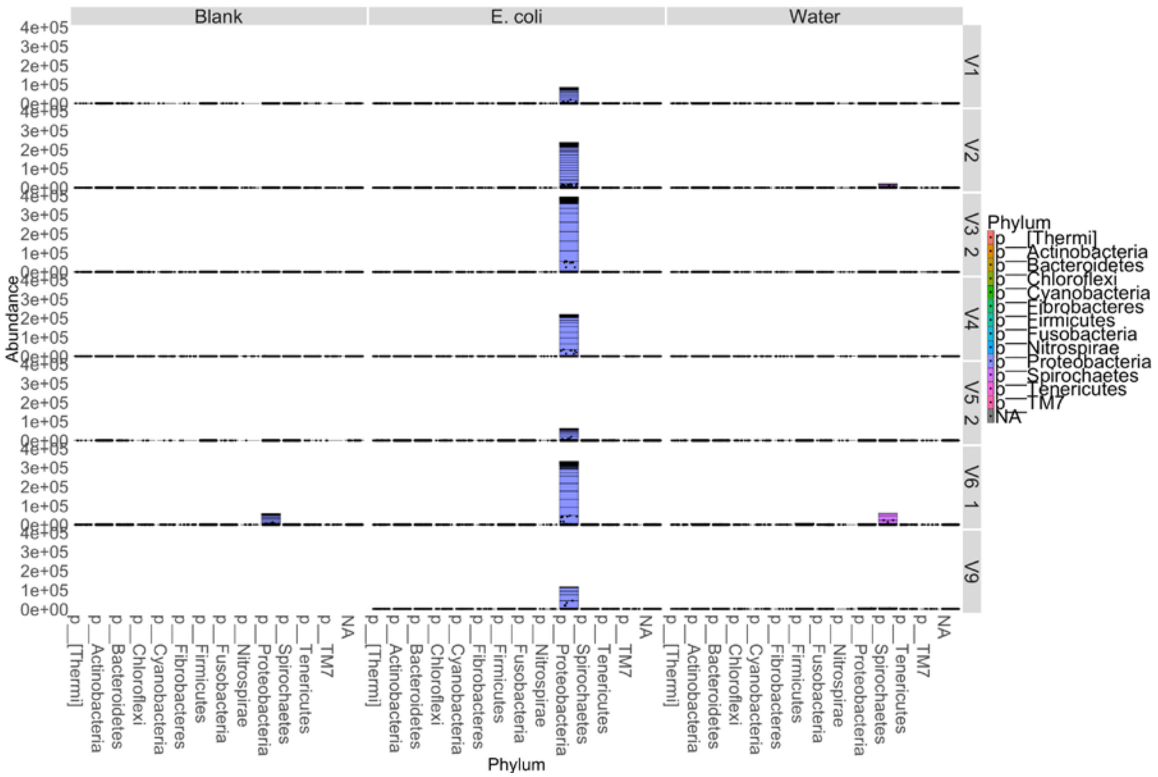

Fig. S2: **Relative abundance of each microbial phyla detected in samples per placental location:** (A) BP (B) PV and (C) FM using V1**,** V2**,** V3_2**,** V4**,** V5_2**,** V6_1**,** and V9 amplicons. Each bar represents total OTUs detected in the sample. Each color represents a different phylum and the horizontal lines within each phylum represent a unique OTU. The labels correspond to the raw data presented as Fasta files in NCBI GenBank.

V1

(A) Basal Plate

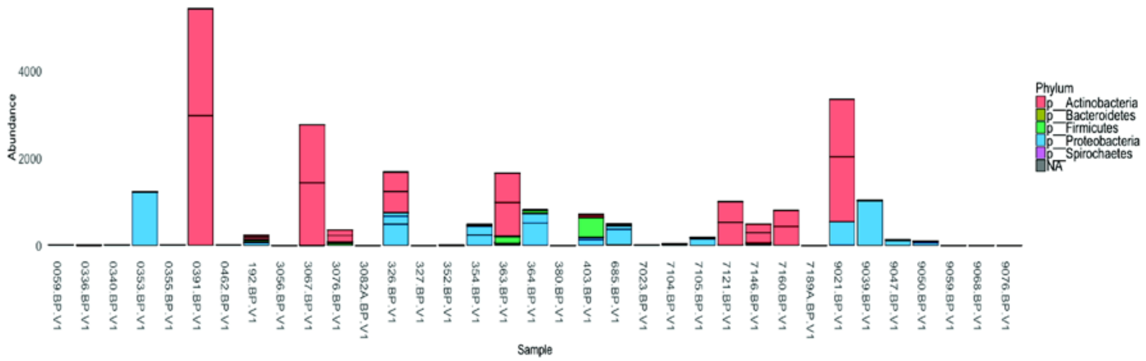

(B) Placental Villus

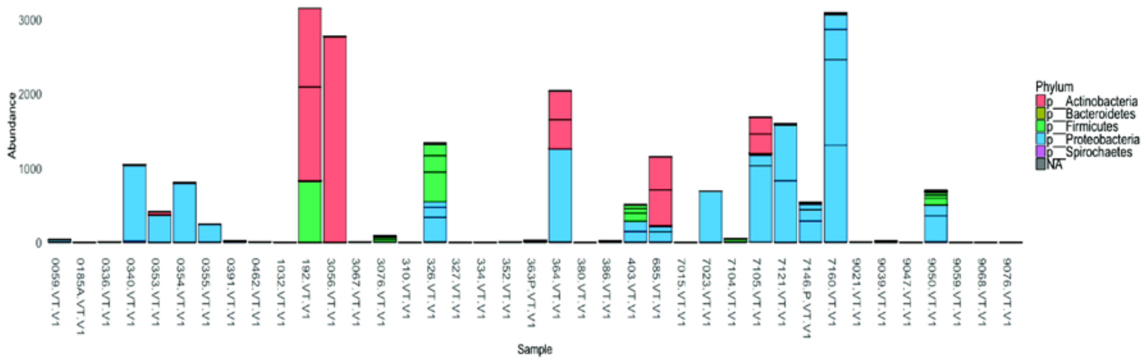

(C) Fetal Membrane

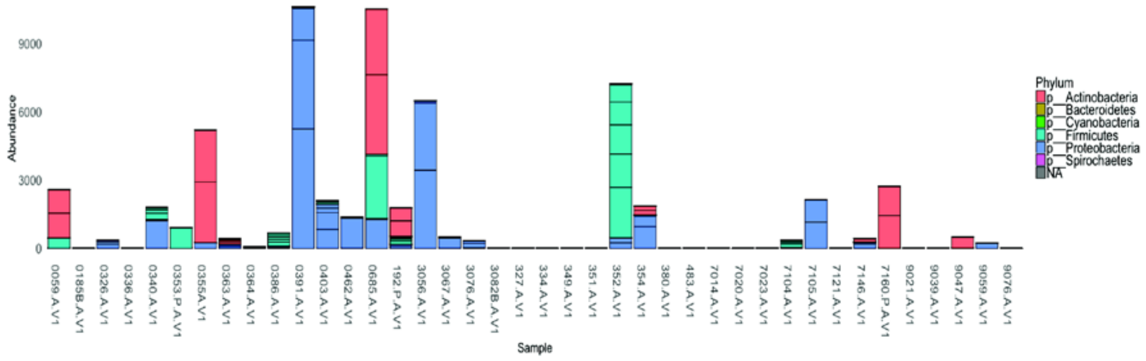

V2

(A) Basal Plate

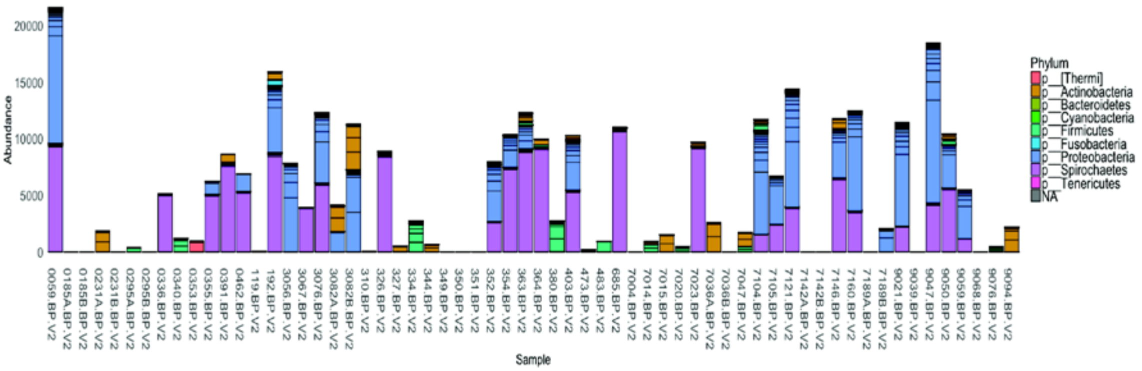

(B) Placental Villus

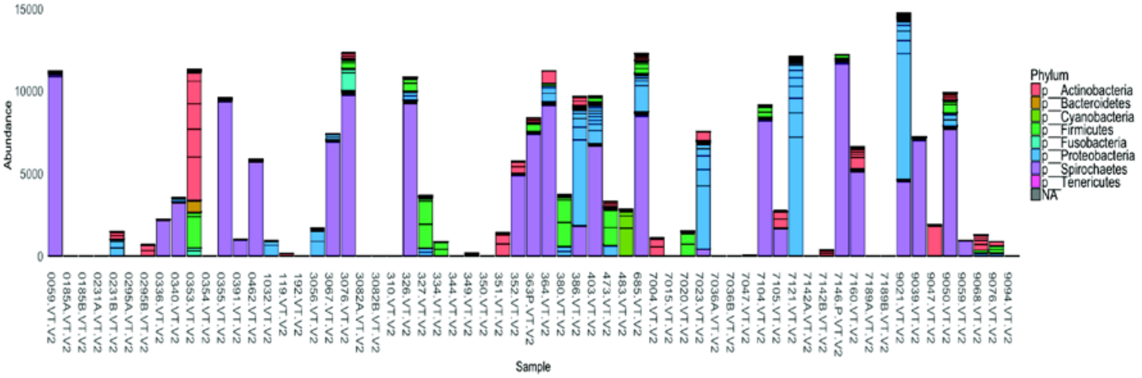

(C) Fetal Membrane

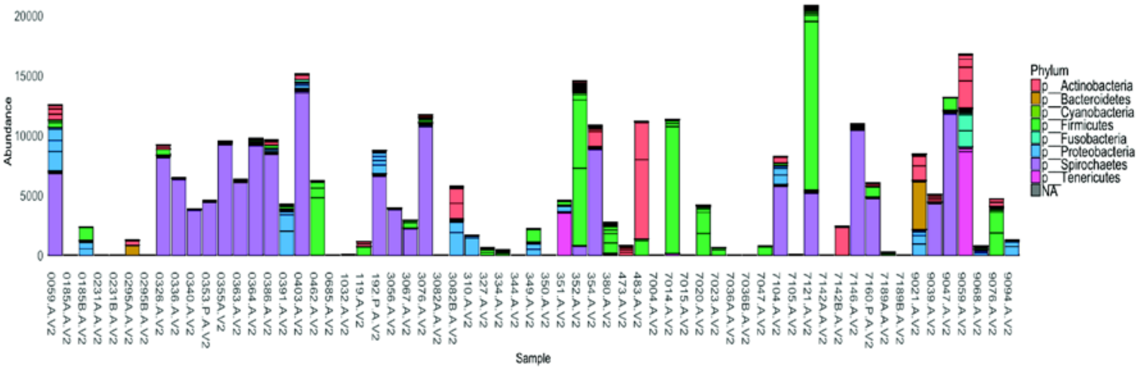

V3_2

(A) Basal Plate

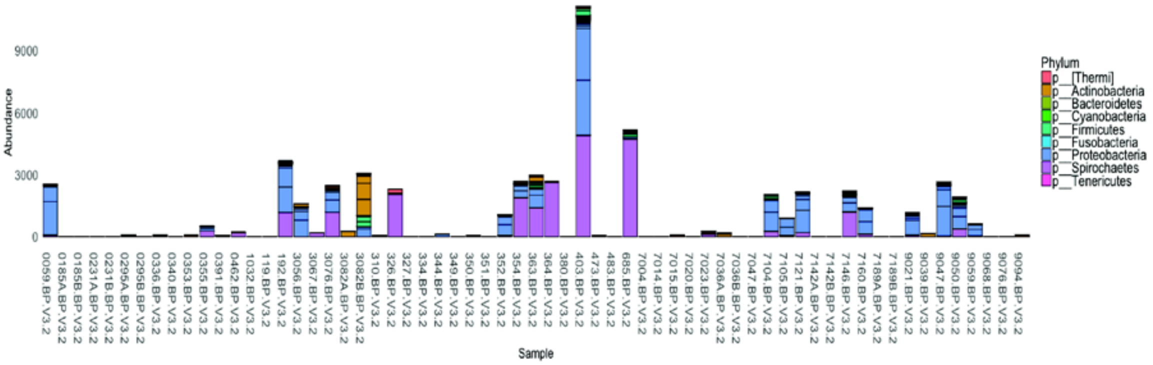

(B) Placental Villus

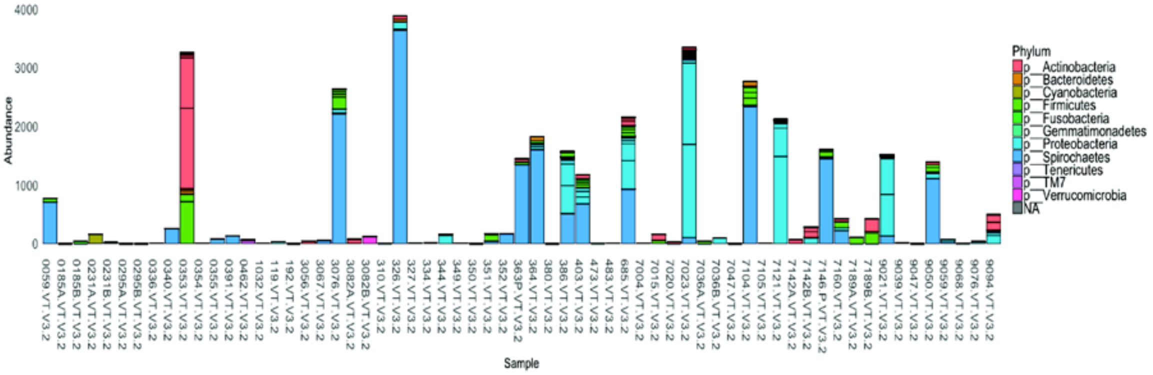

(C) Fetal Membrane

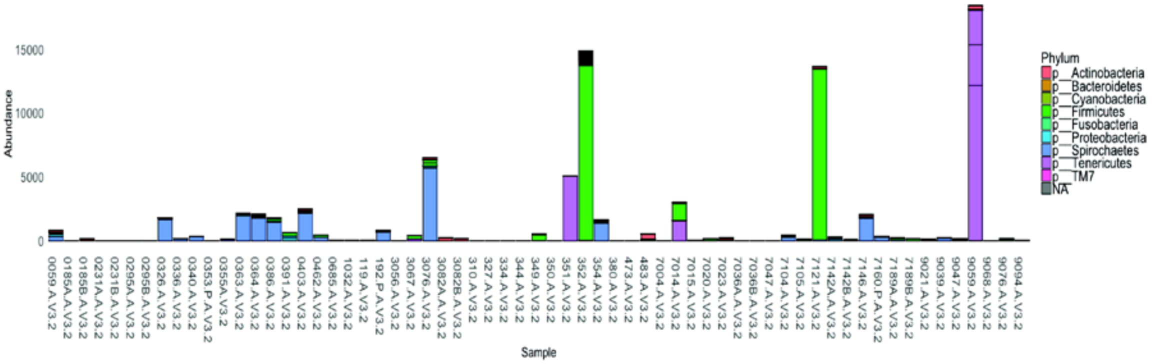

V4

(A) Basal Plate

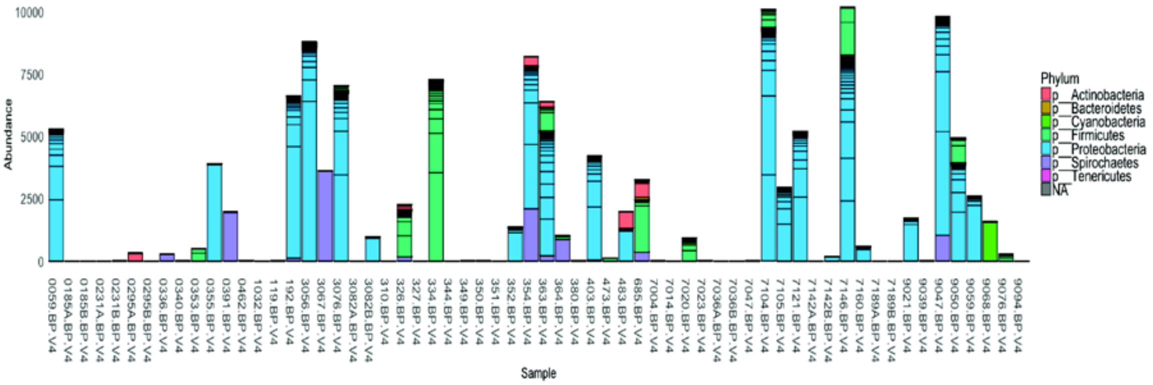

(B) Placental Villus

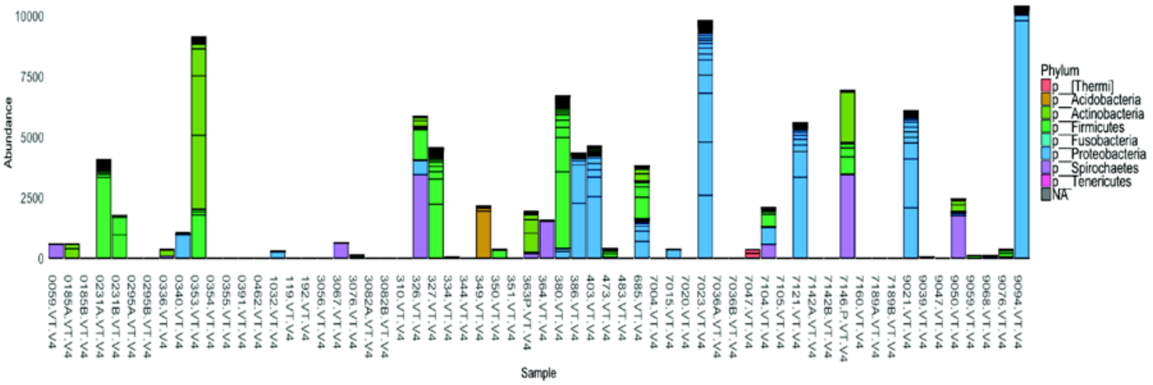

(C) Fetal Membrane

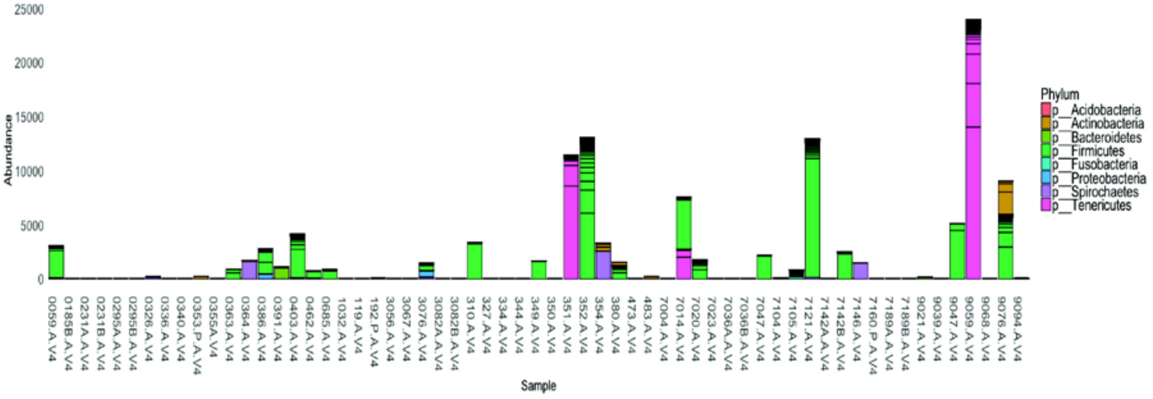

V5_2

(A) Basal Plate

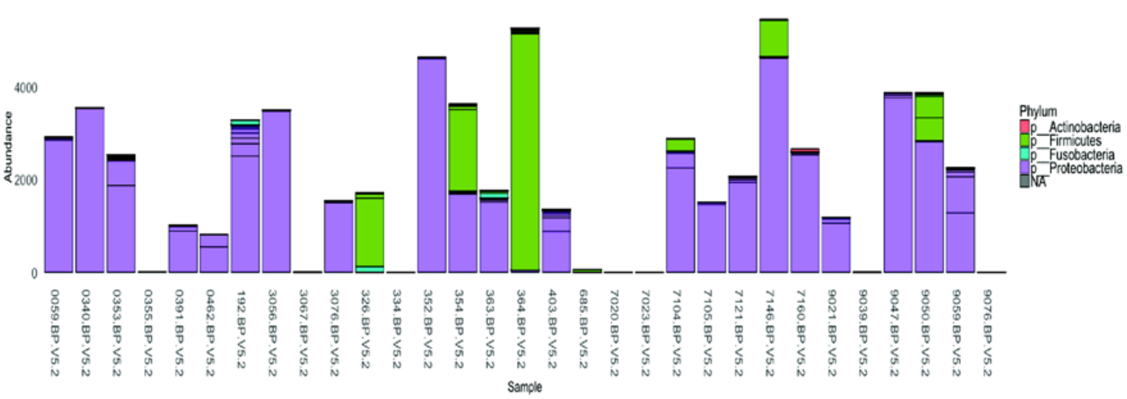

(B) Placental Villus

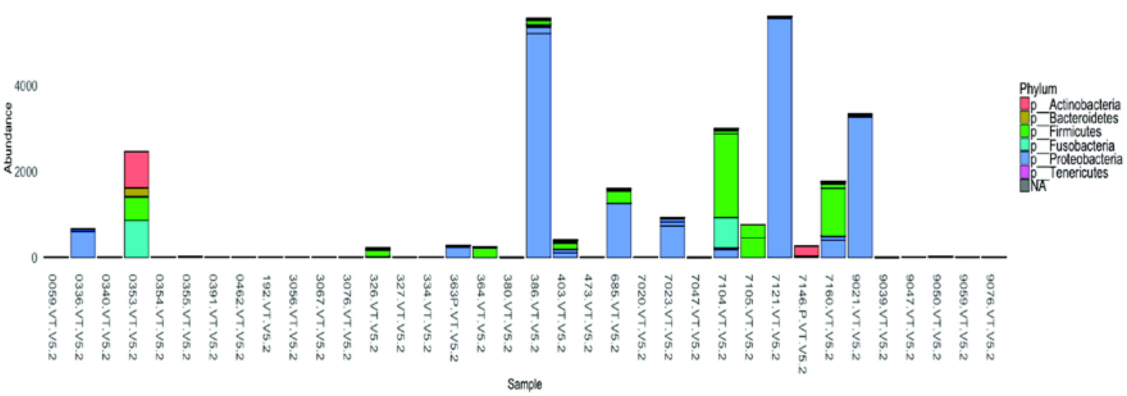

(C) Fetal Membrane

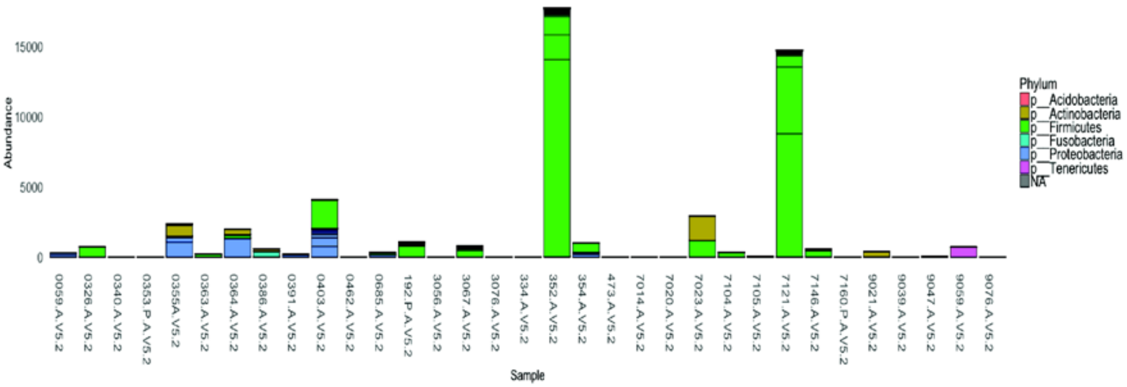

V6_1

(A) Basal Plate

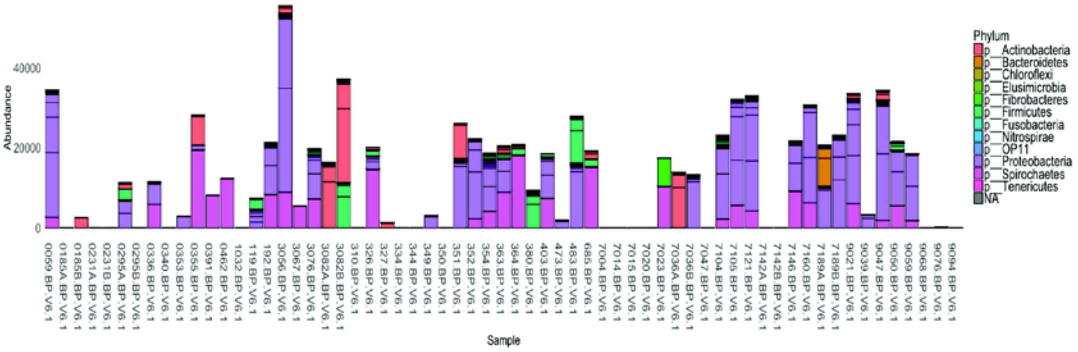

(B) Placental Villus

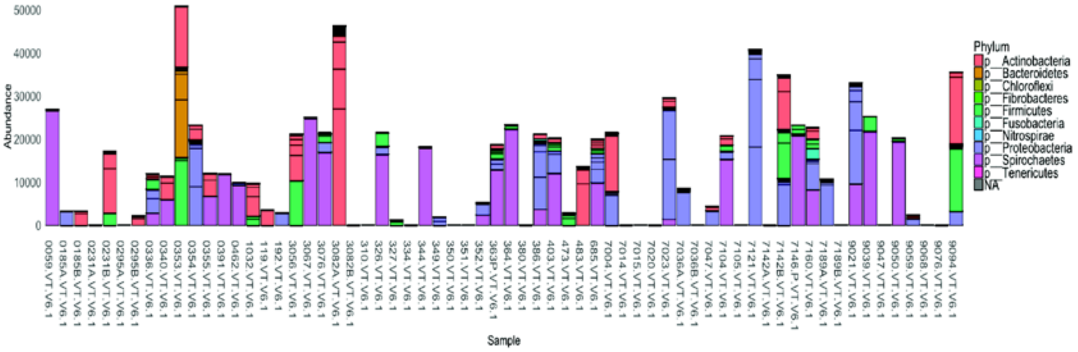

(C) Fetal Membrane

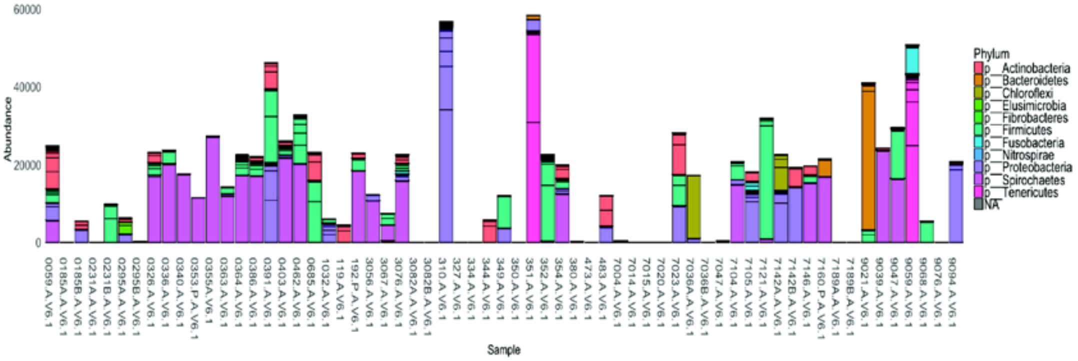

V9

(A) Basal Plate

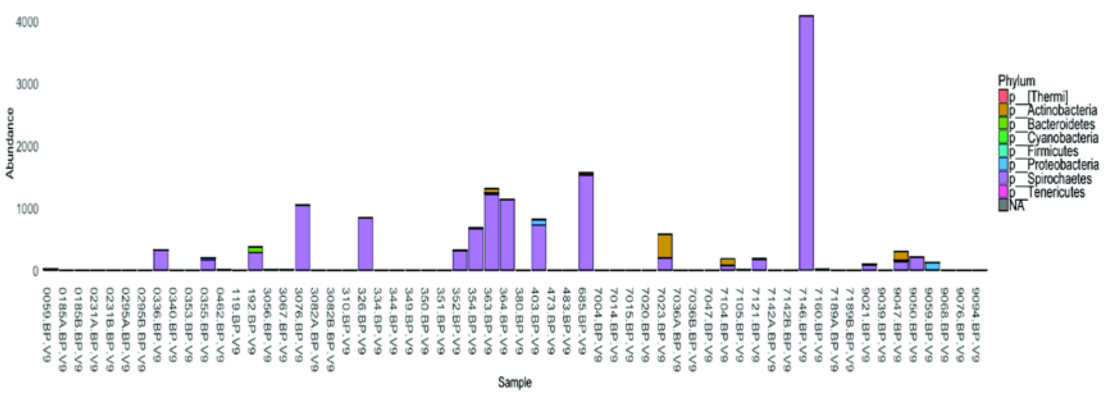

(B) Placental Villus

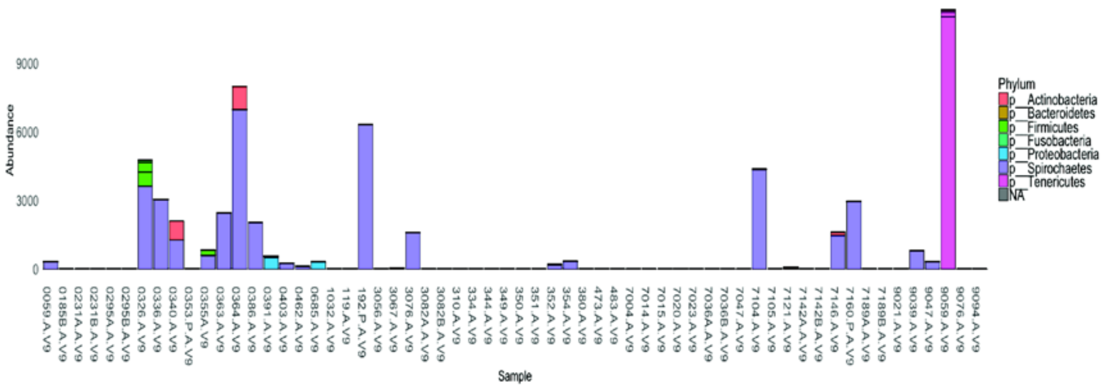

(C) Fetal Membrane

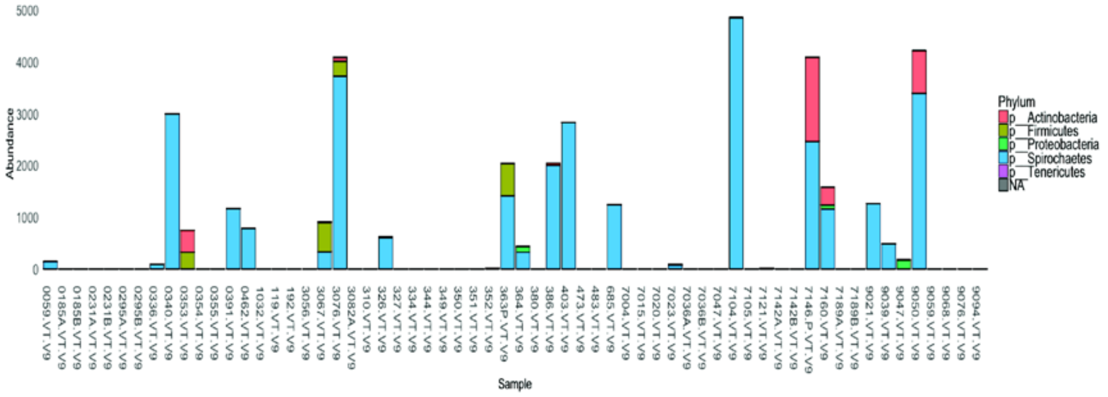

Fig. S3. **Multiple Rarefaction Curves of V4 samples.** The expected Shannon diversity (*H*), which assesses the even-ness and abundance of OTUs, per sample (each color) is shown at multiple sequencing depths (at a step-size of 10, starting from 10 to 300 reads).

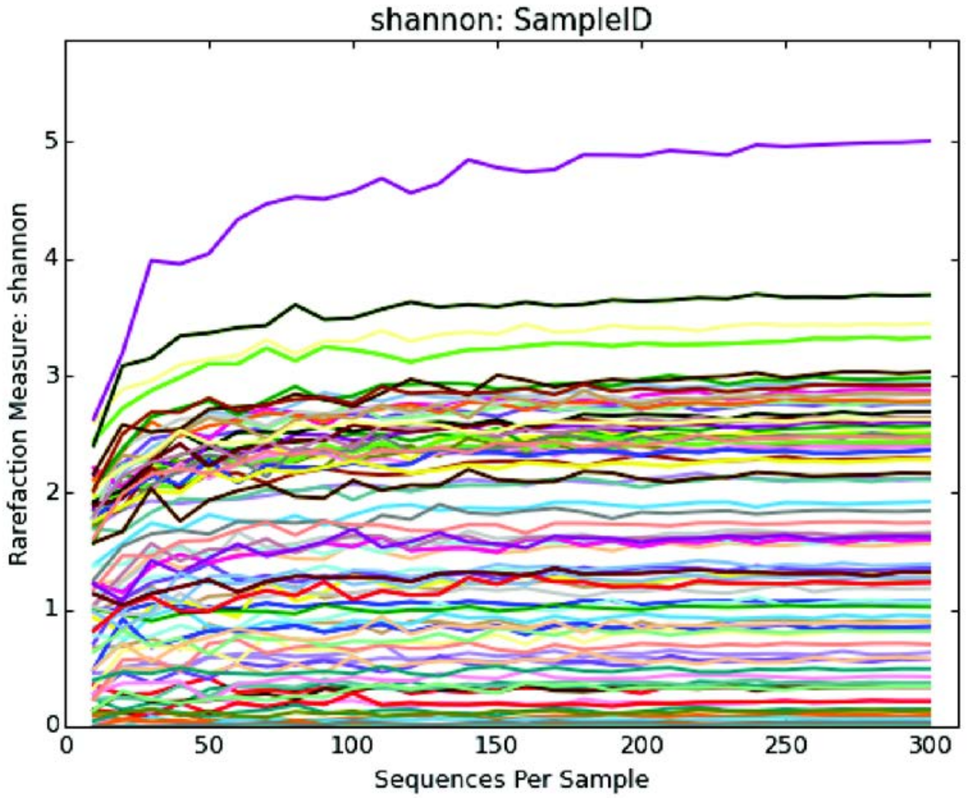

Fig. S4**: qPCR analysis of placental sample and controls for a given run:** Using the logarithmic standard curve generated from one round of qPCR, copy number/µl of placental samples meeting stringent filtering criteria (rarefied to 300 OTUs, no singletons, >34 copies/µL copy number) are highly significantly different from negative controls (p = 0.005 by Mann-Whitney test). p=* <0.05; ** <0.005

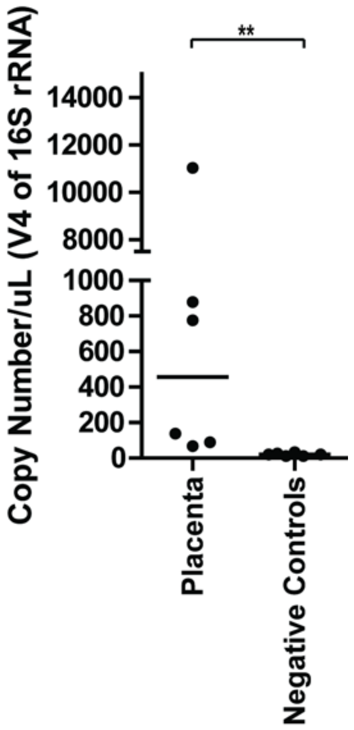

Fig. S5: **Multidimensional scaling (MDS) plot showing relationships between microbial profiles at placental locations using random_tree in the *R* Phyloseq package.** Samples within the BP, PV, and FM were analyzed. Distance measurements are based on Bray-Curtis, unweighted UniFrac, and weighted UniFrac distance metrics (PERMANOVA, Bray: P = 0.001, weighted UniFrac: P = 0.001, weighted UniFrac: P = 0.001).

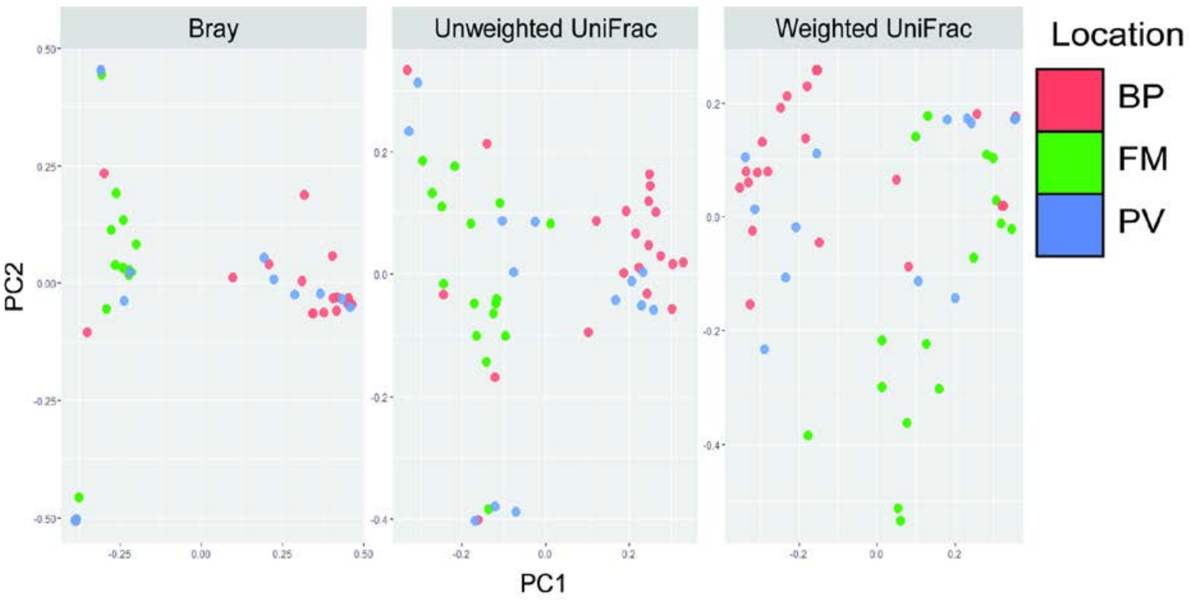

Fig. S6: **qPCR validation of *Ralstonia Insidiosa presence* in placentas using *R.i*-specific primers**

qPCR using *R.i*-specific primers confirmed *R.i* amplification in Basal Plate (BP) samples positive for *R.i* based on species-specific analysis. Each bar represents mean ± SEM Cq value of each sample run in triplicate. Negative controls [Blanks (N=3), molecular biology grade water (N=1)], and samples that did not detect this particular taxa during sequencing; positive control [*E. coli*)], did not amplify *R.i.*

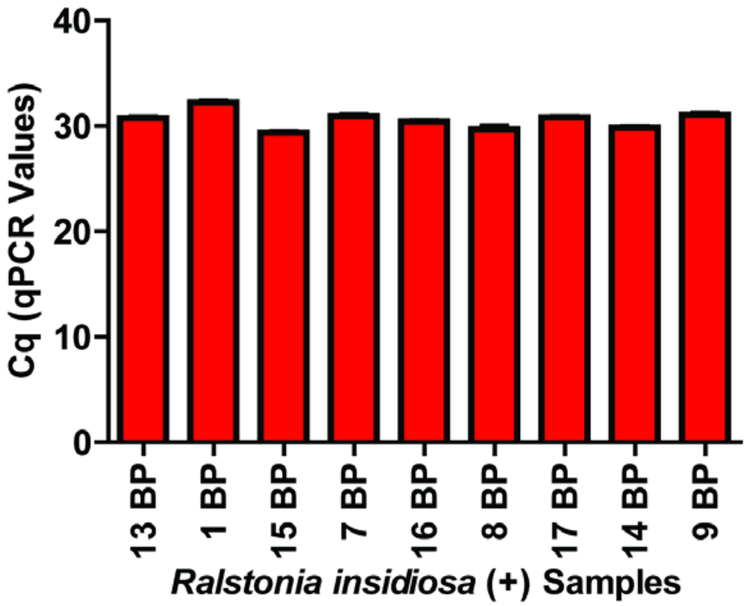

Supplement: Supplementary file 1 — Supplemental material [file 41598_2017_11514_MOESM1_ESM.pdf]
